# Perspectives for Combining Viral Oncolysis With Additional Immunotherapies for the Treatment of Melanoma

**DOI:** 10.3389/fmolb.2022.777775

**Published:** 2022-04-14

**Authors:** Otto Luiz Dutra Cerqueira, Fernanda Antunes, Nadine G Assis, Elaine C Cardoso, Maria A Clavijo-Salomón, Ana C Domingues, Nayara G Tessarollo, Bryan E Strauss

**Affiliations:** ^1^ Centro de Investigação Translacional em Oncologia (CTO)/LIM, Instituto do Câncer do Estado de São Paulo (ICESP), Faculdade de Medicina da Universidade de São Paulo (FMUSP), São Paulo, Brazil; ^2^ Department of Pediatrics, Faculdade de Medicina da Universidade de São Paulo (FMUSP), São Paulo, Brazil

**Keywords:** immunotherapy, gene therapy, adenovirus, melanoma, checkpoint inhibitors, CAR-T cells, p53, interferon

## Abstract

Melanoma is the deadliest type of skin cancer with steadily increasing incidence worldwide during the last few decades. In addition to its tumor associated antigens (TAAs), melanoma has a high mutation rate compared to other tumors, which promotes the appearance of tumor specific antigens (TSAs) as well as increased lymphocytic infiltration, inviting the use of therapeutic tools that evoke new or restore pre-existing immune responses. Innovative therapeutic proposals, such as immune checkpoint inhibitors (ICIs), have emerged as effective options for melanoma. However, a significant portion of these patients relapse and become refractory to treatment. Likewise, strategies using viral vectors, replicative or not, have garnered confidence and approval by different regulatory agencies around the world. It is possible that further success of immune therapies against melanoma will come from synergistic combinations of different approaches. In this review we outline molecular features inherent to melanoma and how this supports the use of viral oncolysis and immunotherapies when used as monotherapies or in combination.

## Introduction

Immunotherapy has revolutionized cancer treatment in unprecedented ways (Mellman et al., 2011). It consists of mobilizing the immune system’s own defenses to recognize and eliminate neoplastic cells, thus reinstating cancer immunosurveillance (Zitvogel et al., 2013). The promising early results generated high expectations and gave hope to many patients to reach long-term remission (Cable et al., 2021). However, only a minority of patients with advanced cancer undergoing this therapeutic modality increase survival in a lasting way (Hegde and Chen, 2020). In this scenario, the use of rational combinations of immunotherapies has been increasing and may link different approaches and technologies in order to improve patient benefit of the treatment (Finck et al., 2020). Oncolytic viruses (OV) are therapeutic tools with the property of not only selectively inducing oncolysis, but also attracting cells of the immune system, activating them and thus mobilizing innate and adaptive antitumor responses (Vähä-Koskela et al., 2007; Russell et al., 2012). This property is suitable for combination with therapies aimed at cell-mediated cytotoxic effect, whether adoptive or naturally intrinsic to the immune system, acting synergistically at different stages of the cancer-immunity cycle (Chen and Mellman, 2013). Here we provide a historical and biological perspective regarding the advent and clinical implementation of immunotherapies, including oncolytic viruses, for the treatment of melanoma and we explore the possible combinations of oncolytic viruses with additional immunotherapy strategies.

## Molecular Alterations in Melanoma That May Serve as Therapeutic Targets

Cancer is a multifactorial disease characterized by heterogeneous subpopulations of cells with different phenotypes and genetic properties leading to uncontrolled proliferation, migration, invasion as well as metastasis and drug resistance. Skin cancer can be classified as basal cell carcinoma, squamous carcinoma and melanoma. Non-melanoma skin cancers account for nearly 98% of skin cancer in the United States (Force et al., 2018). Although melanoma is the least frequent type of skin cancer, it is the deadliest with steadily increasing incidence worldwide during the last few decades, especially in Caucasian populations (Siegel et al., 2020). The latest data released by Global Cancer Observatory (GLOBOCAN) estimated more than 280,000 new cases of skin melanoma (1.6% of all cancers) with nearly 60,000 deaths in 2020 (Global Cancer Observatory–https://gco.iarc.fr/). Melanoma arises from malignant transformation of melanocytes, melanin-producing cells found in the epidermal skin layer, due to mutagenic damage that activates many oncogenes and inactivates tumor suppressor genes (Kobayashi et al., 1998; Cancer Genome Atlas, 2015; Hayward et al., 2017). Some studies demonstrated that incidence of skin cancer (non-melanoma and melanoma) is inversely related to skin pigmentation, with higher risk and incidence in individuals with fair skin along with the presence of nevi and freckles. Furthermore, a family history of melanoma, immunosuppression related to organ transplantation, and HIV or HPV infection also increase the predisposition to this neoplasm (Veierod et al., 2010; Force et al., 2018).

One of the main risk factors for the development of melanoma is intermittent excessive exposure to solar ultraviolet (UV) radiation, being particularly harmful when it occurs in childhood. In addition, there is growing evidence that exposure to artificial UV radiation through the use of artificial tanning chambers increases the propensity to develop melanomas (Veierod et al., 2010; Sun et al., 2020). UV radiation induces DNA damage directly (DNA photoproducts) or through ROS production that indirectly causes oxidative DNA damage, leading to DNA mutations and alterations in the transcriptional profile, resulting in dysregulation of several tumor suppressor genes and oncogenes (Kvam and Tyrrell, 1997; Anna et al., 2007). Moreover, UV radiation can also downregulate cutaneous immunity by apoptosis of epidermal immune cells (Langerhans cells) and inhibition of antigen presentation together with release of immunosuppressive cytokines, favoring tumor development and progression (Fisher and Kripke, 1982; Shreedhar et al., 1998). The UV-induced DNA damage response is modulated by the tumor suppressor gene *TP53* that can be found downregulated, contributing to UV-mediated mutagenesis in non-melanoma and melanoma skin cancer (Smith et al., 2000; Decraene et al., 2001). In addition, UV exposure can alter *TP53*, resulting in cooperation with *BRAF* mutations to induce melanoma (Viros et al., 2014).

Sequencing studies revealed the genetic landscape of cutaneous melanoma and classified them into four subgroups: mutant *BRAF*, mutant *NRAS*, mutant *NF1* and triple-wild type (Cancer Genome Atlas, 2015; Hayward et al., 2017; Zhou et al., 2019). In another study, the whole genome sequence analysis of melanoma samples also found mutations in other genes, such as *TERT*, *TP53*, *CDKN2A* and *CDKN2B*. Some of these mutations as *BRAF*, *NRAS* and *TERT* are also found in benign lesions whereas *CDKN2A*, *TP73* and *PTEN* are observed only in invasive melanoma (Amaral et al., 2017; Consortium, 2020). Other mutations less frequently found, especially in melanomas missing heritability, are *BPA1*, *POT1*, *ACD* and *TERF2IP* (Potjer et al., 2019).

*BRAF* mutations are highly prevalent in melanoma and found in 40–60% of cultured primary melanoma cells but are not sufficient for melanoma progression and development since they are found in benign nevi (Pollock et al., 2003; Tschandl et al., 2013). The most frequent oncogenic mutation for *BRAF* in melanomas is the substitution of amino acid valine for glutamic acid at position 600 (V600 E), representing 70–90% of *BRAF* mutations. Other *BRAF* mutations, although less frequent, can be found in melanoma, including V600K, V600R, V600D for example (Rubinstein et al., 2010; Long et al., 2011; Lovly et al., 2012). Mutations in *BRAF* are not related to UV radiation exposition as 30–60% of patients without chronic sun-induced damage have been identified with somatic BRAF mutation (Curtin et al., 2005; Brash, 2015). These mutations have important clinical significance since mutated BRAF protein is active as a monomer instead of dimer and the monomer conformation is the target for the binding of BRAF inhibitors, such as vemurafenib, dabrafenib and encorafenib, used in melanoma therapy (Czarnecka et al., 2020). Moreover, the presence of *BRAF* mutations (*BRAF* (+)), despite not impacting recurrence-free survival from diagnosis of primary melanoma (stage I/II) to metastases development (stage IV) compared to *BRAF* WT patients, they do have a negative impact on median overall survival (OS) of patients who are newly diagnosed, untreated and with metastatic disease, since in *BRAF* (+) patients the OS is 5.7 months and for *BRAF* WT it is 8.5 months (Long et al., 2011). BRAFV600 E mutation resulted in altered BRAF protein conformation, increasing its kinase activity, leading to constitutive MAPK pathway activation, resulting in uncontrolled proliferation, cell survival and immune evasion which contribute to melanoma growth (Yang et al., 2019). The MAPK pathway is also activated by *NRAS* mutations that are frequently found in several tumor types and in 15–20% of melanoma patients but not concomitant with *BRAF* mutations (Wan et al., 2004; Chiappetta et al., 2015). Moreover, 15% of melanomas have *NF1* mutations with loss of function that also result in MAPK hyperactivation (Wan et al., 2004; Krauthammer et al., 2015). Deregulation of RAS/MAPK/ERK pathway is found in nearly all melanomas (Hayward et al., 2017). The signaling pathway RAS/MAPK/ERK impacts more than 50 transcription factors involved in the regulation of genes that control cell growth, division, proliferation and differentiation (Molina and Adjei, 2006). The pathway is activated by cytokines, growth factors and hormones which interact with a membrane tyrosine kinase receptor, inducing its phosphorylation and leading to signal transduction by subsequent phosphorylation of a series of proteins from RAS, RAF (ARF, BRAF, CRAF), MEK (MEK1 and MEK2) and MAPK/ERK family. The activated ERK goes to the nucleus where it activates transcription factors such as cMyc and CREB by phosphorylation (Molina and Adjei, 2006). The activated MAPK pathway also has an immunosuppressive effect due to downregulation of tumor antigens and decreased recognition by immune cells together with upregulation and infiltration of immunosuppressive cells after cytokine secretion (Ott and Bhardwaj, 2013; Yang et al., 2019).

Another important pathway commonly upregulated in melanomas is that of PI3K/AKT/mTOR which regulates cell proliferation, cellular response during stress and quiescence, contributing to tumor growth, metastasis and angiogenesis induction in melanomas (Porta et al., 2014). The most common mutations contributing to this activation are found as upregulation of the oncogene *NRAS* (15–20%) and loss of function or expression of the tumor suppressor *PTEN* (20–30%), yet these are largely mutually exclusive events (Hocker and Tsao, 2007; Aguissa-Toure and Li, 2012). On the other hand, *PTEN* loss can occur concomitantly with *BRAF* mutations, resulting in activation of RAS/RAF/MAPK and PI3K/AKT pathways (Tsao et al., 2004; Goel et al., 2006). Activated AKT phosphorylates several proteins, including antiapoptotic proteins (XIAP, BAD, BIM), MDM2, p21 and many others, allowing survival and progression of melanoma cells together with apoptosis inhibition (Madhunapantula et al., 2011). Interestingly, *PTEN* loss is also related to immunosuppressive properties such as lower sensitivity to T cell mediated cell death and reduced infiltrations of T cell infiltration in the tumor site, contributing to melanoma immune resistance (Peng et al., 2016).

**FIGURE 1:**
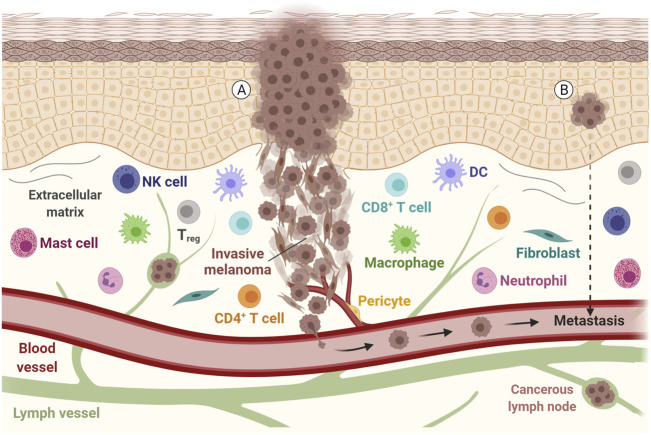
Representation of a melanoma tumor during invasion and metastasis**.** Tumor cells with epithelial and mesenchymal-like morphology are shown, along with other components of the tumor microenvironment, such as the extracellular matrix, immune cells and fibroblasts. The communication between these components, with lymph vessels, blood vessels and tumor cells may allow the tumor to spread. **(A)** Metastatic disease is found in patients with clinically identified proliferating melanoma, **(B)** but also in patients with an undetected source of tumor cells or evident primary lesion. DC, Dendritic cell; NK, Natural killer; T_reg_, Regulatory T cell. Adapted from “Melanoma Staging”, by BioRender.com (2021). Retrieved from https://app.biorender.com/biorender-templates.

Melanomas bearing BRAFV600 E mutations commonly also have altered MITF expression and activity (Levy et al., 2006). The *MITF* gene encodes a central regulator of melanocyte differentiation, development and function, besides several biological processes such as DNA repair, senescence, cell metabolism, survival, differentiation, proliferation and metastases formation (Goding and Arnheiter, 2019). MITF can be employed as a diagnostic marker for tumors from melanocytic origin, however, with different levels of expression correlating with distinct behavior of malignant cells (Levy et al., 2006). High MITF expression is associated with highly proliferative and poorly invasive phenotype while low MITF expression correlates with a slowly proliferative and highly invasive profile. *In vivo* studies demonstrated that although different in MITF expression, both phenotypes can establish tumors when inoculated into nude mice but with the invasive phenotype requiring a longer period to develop palpable tumors (Hoek et al., 2008; Vachtenheim and Ondrusova, 2015). However, both invasive and proliferative phenotypes can be present simultaneously since melanoma progression is not associated just with differential gene expression (Harbst et al., 2012; Cancer Genome Atlas, 2015), but also with dynamic transcription signature plasticity which contributes to tumor metastization due to the adaptive response to the tumor microenvironment (Roesch et al., 2016) (Figure 1A) Also reported for melanoma is the P29 S mutation in the *RAC1* gene that is found in approximately 3% of melanomas but in almost 20% of patients resistant to BRAF inhibitors (Watson et al., 2014). RAC1 is involved in cellular adhesion, motility and differentiation, and the consequences of mutation in this gene are melanocytic to mesenchymal phenotype, increased tumor size and presence of metastasis (Lionarons et al., 2019).

The expression of immune-related genes also correlates with prognosis and response to melanoma immunotherapy as demonstrated after analysis of 45 patients submitted to anti-CTLA-4 therapy that had tumors with increased expression of genes related to immune signature (Ji et al., 2012). The interferon pathway is one of the key players in the response to immunotherapy and the type I and II interferons are mainly responsible for antitumor response due to increased immune recognition and apoptosis induction in tumor cells (Benci et al., 2016; Gao et al., 2016; Sharma et al., 2017). Many tumors have impaired interferon signaling resultant from alterations in regulatory genes such as loss of *IFNGR1*, *IRF1*, *JAK2* and amplification of *SOCS1* and *PIAS4* (Gao et al., 2016). Analysis of the TCGA dataset and studies *in vitro* and *in vivo* showed that nearly 30% of melanoma samples present mutations in the interferon signaling pathway, which is associated with shorter overall survival (Gao et al., 2016). Moreover, increased expression of interferon-related genes (e.g., *CXCL4*, *CXCL5*, *CXCL10*, *ID O 1*, *IRF1*, *STAT1* and others) was associated with benefit from anti-PD-1 and anti-CTLA-4 immunotherapy in melanoma patients (Ji et al., 2012; Gide et al., 2019). On the other hand, the suppression of the interferon pathway is associated with poor response to immunotherapy protocols due to immune evasion (Jerby-Arnon et al., 2018).

Melanoma patients’ deaths are mostly associated with distant metastasis development, showing a 5-years survival rate of around just 20% (Bomar et al., 2019). However, some melanoma patients have metastatic disease without evident primary lesion (Figure 1B) and in this case, the disease development is associated with immunoediting mechanisms together with loss of immunohistochemical melanocytic markers like S100 protein, HMB-45, Melan-A, SOX10 and MITF (Gyorki et al., 2013; Bankar et al., 2015). The exhaustion of the immune system and immune evasion are among the key factors that enable melanoma growth and metastasis formation (Passarelli et al., 2017; Motofei, 2019). Moreover, *BRAF* mutations are present in about 50–60% of metastatic melanoma cases (Zaman et al., 2019). Indeed, studies have revealed that cutaneous melanoma has a high mutation rate compared to other common tumors, with a mean tumor mutation burden (TMB) of over 20 mutations per megabase, one of the highest TMB among solid tumors (Cancer Genome Atlas, 2015; Zhang et al., 2016). Malignant melanoma is highly genetically heterogeneous, with prevalence of somatic mutations in primary tumors and metastatic lesions that also acquire numerous mutations during their formation (Hoek et al., 2006; Swick et al., 2012).

## Melanoma Antigens and Immunogenicity

The immune system has the inherent property to distinguish self from non-self-antigens (Yarchoan et al., 2017). Despite the fact that tumor cells arise from healthy tissues, hence self, the ability of the immune system to recognize them is based on an important concept: neoantigens (also referred to as neoepitopes), which arise from tumor-specific mutations (Alexandrov et al., 2013). Since melanomas have a high mutational burden, which is reflected in the higher levels of neoantigens, they are more likely to promote immune response and be recognized by the immune system (Maleki Vareki, 2018). Described immunologically as “hot”, these tumors offer a huge repertoire of potential targets for T cells that, in principle, reflect a greater inflammatory infiltrate (Maleki Vareki, 2018). This point has been extensively explored in several approaches in cancer treatment, such as cancer vaccines against neoantigens and adoptive T cell transfer, which can be combined with immunotherapy targeting T cell inhibitory receptors, including cytotoxic T-lymphocyte associated antigen (CTLA)-4 and programmed cell death (PD)-1 (Peng et al., 2019). The clinical benefits of immune checkpoint inhibitors are often observed in high mutational load tumors, which may be related to the presence of tumor associated antigen-specific T cells (Banchereau and Palucka, 2018).

The antitumor immune response is mainly mediated by the adaptive immune system, especially the tumor-infiltrating lymphocytes (TILs) (Lugowska et al., 2018) that can recognize through the T cell receptors (TCRs) antigenic peptides presented *via* major histocompatibility complex molecules (Durgeau et al., 2018). In the case of human melanomas, the high degree of TILs and, more specifically, cytotoxic T cell infiltration, together with elevated expression of checkpoint receptors make melanoma patients more likely to respond successfully to immunotherapy (Galon and Bruni, 2019).

Antigen targets of immunotherapy can be divided into tumor associated antigens (TAAs), which include the cancer testis antigens (CTAs), and tumor specific antigens (TSAs) (Aurisicchio et al., 2018). TAAs include proteins encoded in the normal genome, usually expressed at low levels, and might be over-expressed in malignant cells. CTAs are normally expressed in testis, fetal ovaries, and trophoblasts, but can also be expressed in cancer cells. Because TAAs and CTAs are found in normal cells, their antigenicity depends on abnormal expression levels and, frequently, their presence in the tumor microenvironment can lead to immunological tolerance. The third class comprises antigens that are not encoded in the normal host genome and are originated by somatic mutations in the coding sequence, creating a unique peptide sequence (Gubin et al., 2015), or by insertion of oncogenic viral genes, such as E6 and E7 encoded by human papillomavirus type 16 that drive oral and cervical tumors (Walboomers et al., 1999).

Many TAAs have been used for years to assist clinical practice. For example, the human epidermal growth factor receptor (HER2) is routinely used for breast cancer prediction and prognosis (Patani et al., 2013). Melanoma TAAs include the type 1 melanoma antigen recognized by T cells (MART-1, also known as Melan-A) and the melanoma-associated antigen (MAGE). In a phase 1/2 clinical trial, a three-dose vaccine strategy using autologous DCs transduced with an adenoviral vector encoding the MART-1 antigen for metastatic melanoma patients showed that at least half of the treated patients had significant MART-1–specific T cell responses (Butterfield et al., 2008). Similarly, in a phase II study DCs were pulsed with a cocktail of melanoma-associated antigens, including MART-1 or MAGE-A1, MAGE-A2, MAGE-A3, gp100 and tyrosinase, and were subcutaneously injected in metastatic melanoma patients, for which 75% had an antigen-specific CTL response. Notably, patients in the vaccinated group with two or more peptide-specific responses had a significantly longer mean survival time (21.9 months) compared to treated patients who had less than two peptide-specific responses (8.1 months) (Oshita et al., 2012). Recently identified potential melanoma biomarkers, in addition to the more than 45 already studied (Belter et al., 2017), include metabolic components, for instance aminomalonic acid and phosphatidylinositol (PI) (Kim et al., 2017), as well as immune-related genes and TCRs (Charles et al., 2020; Huang et al., 2020). Although the clinical trials using TAA have shown initial immune response, most of them have failed to demonstrate durable beneficial effects. The main reasons are the lower TCR affinity to TAA and peripheral tolerance of TAA-reactive T cells (Melero et al., 2014).

Beyond TAAs, TSAs are attractive targets for immunotherapy. These neoantigens are expressed only in cancer cells and can be recognized by TCR with high affinity. Neoantigen-specific T cells are not subject to central and peripheral tolerance and, consequently, their activation leads to a lower induction of autoimmunity (Yarchoan et al., 2017). As a result, the antitumor immune responses to TSAs are more robust as compared to TAAs. An important advance in the understanding of TSAs and immune response was published in 2005 by Wölfel and colleagues. The authors found that the T cells of a patient were reactive against five mutated epitopes and the immunoreactivity against melanoma neoantigens predominated over the response to TAAs (Lennerz et al., 2005). In addition, Rosenberg’s group showed that the adoptive transfer of *ex vivo*–expanded TILs reactive against two neoantigens into a melanoma patient promoted complete tumor regression. All these studies support the role of neoantigens in the natural antitumoral T cell response (Zhou et al., 2005).

Besides its high TMB (Lawrence et al., 2013) and despite most human melanomas having a mutational load above 10 somatic mutations per megabase of coding DNA, which are generally sufficient to lead to the formation of neoantigens, T cell reactivity is not always observed (Linnemann et al., 2015). Recent studies revealed that both TMB and PD-L1 are not effective biomarkers for identifying patients who will have clinical benefit from checkpoint inhibitor therapy. An important point that may be considered is the clonality of neoantigens. Some evidence suggested that a minimum quantity of cells is required to generate T cell-mediated immune rejection (Gejman and Chang, 2018) and subclonal neoantigens are not presented by every cancer cell, so they are less effective in immune control of disease (Mcgranahan and Swanton, 2019). Furthermore, the majority of neoantigens are considered passenger events and, usually, their loss during tumor progression may be tolerated. However, when the mutations occur in genes required for tumor cell survival (such as cancer driver genes and genes required for cancer cell viability) and these genes are retained despite the events of copy number loss or transcriptional repression through methylation, the neoantigens are considered as essential neoantigens. Due to positive selection, these high-quality neoantigens cannot be repressed or deleted during tumor progression. Thus, both the quantity and the quality of neoantigens, more emphatically the quality, may explain why some patients are good responders to immunotherapy and others are not (Mcgranahan and Swanton, 2019).

However, the study of neoantigens has encountered barriers due to the lack of effective tools for their identification. In 2012, using a combination of next generation sequencing and algorithms for predicting the binding of peptides to MHC class I and class II molecules, Castle and coworkers identified TSAs in B16-F10 mouse melanoma cells (Castle et al., 2012). In parallel, exome sequencing and high-throughput MHC tetramer screening showed higher expansion of pre-existing T cells specific for tumor neoantigens in a human melanoma patient after treatment with checkpoint blockade immunotherapy (Van Rooij et al., 2013). To improve the adoptive transfer of T cells, Lu et al. genetically engineered autologous T cells to have neoantigen-specific TCRs. Isolated TILs were cultured and screened for the identification of neoantigen-reactive T cells to be further co-cultured with peptide-pulsed APCs. The single-cell RNA-sequencing allowed the identification of different neoantigen-specific TCRs, for instance a mutated KRAS-specific TCR, which could be successfully transduced into autologous T cells and recognize the specific neoantigens presented by the donor APCs (Lu and Robbins, 2016). The use of genomics and bioinformatics approaches in both mouse and human studies supported the rapid identification of mutant proteins expressed exclusively in cancer cells that act as neoantigens compared to conventional antigen-cloning approaches (Gubin et al., 2015) and highlight the potential of personalized cancer vaccines targeting neoantigens.

In a phase I study, 10 patients with stage IIIB/C or IVM1a/b melanoma were vaccinated with 13–20 personalized neoantigens peptides synthesized from sequencing of the tumors. After 20–32 months from vaccination, four patients with stage III disease were recurrence-free. Two patients with lung metastases had a complete response with the anti-PD-1 antibody, indicating the expansion of neoantigens specific T cells (Ott et al., 2017). Similar results were found by Sahin and colleagues who used personalized RNA-based ‘poly-epitope’ vaccine in 13 patients with stage III or IV melanoma. Each patient developed an immune response against at least three mutations. One patient with relapse and progressive disease at the time of vaccination presented a complete response after administration of anti-PD-1 antibody and eight continued disease-free 12–23 months later (Sahin et al., 2017). Both studies revealed that immune response was generated by CD4^+^ T cells and the vaccination provided the expansion of the neoantigen-specific T cells (Li L. et al., 2017). These studies confirm the potential of the immunogenic melanoma neoantigens and open novel possibilities for approaches using neoantigens as vaccinogenic agents associated with diverse delivery vehicles such as synthetic and biological nanoparticles and adenoviral vectors.

Although neoantigens are a promising strategy, the non-synonymous mutations that will originate the mutated protein depend on several factors that need to be present; the sequence with the mutation must be translated into protein, the mutated protein must be processed, and the peptides must be presented by MHC molecules. At the end of the process, the affinity between the mutated peptide and the patient’s MHC molecules will determine recognition by the TCR (Schumacher and Schreiber, 2015). All of these processes are susceptible to complications that can alter the TCR-MHC binding, contributing to tumor escape from the immune system and also confounding *in silico* approaches for the prediction of the most effective neoantigens.

## Current Immunotherapeutic Strategies and Challenges

More than 10 different drug types have been approved by the FDA for the treatment of melanoma, including dacarbazine chemotherapy, BRAF and MEK-targeted therapy, recombinant interferon alpha-2b and IL-2, immune checkpoint inhibitors (ICIs), and oncolytic viral therapy (T-VEC). These strategies, together with radiation therapy and surgery comprise the clinical arsenal against primary and metastatic melanoma (Garbe et al., 2020; Jenkins and Fisher, 2020). During the past 20 years, ICIs (commonly referred to as immunotherapy) have taken on a leading role and occupied center stage in the melanoma treatment scene. Extraordinary results were achieved with anti-CTLA-4, anti-PD-1 and anti-PD-L1 in a time when targeted therapy only offered reasonable short-term, but poor long-term, overall survival (Ribas et al., 2012; Spagnolo et al., 2016; Li X. et al., 2017; Karlsson and Saleh, 2017). Their success turned ICIs into the standard of care for advanced melanoma, after surgery, demonstrating that when the immune system is activated properly, it may lead to durable long-term responses.

In terms of improved efficacy and reduced toxicity, studies have found that anti-PD-1 (Nivolumab or Pembrolizumab) is superior to anti-CTLA-4 (Ipilimumab) (Li X. et al., 2017; Karlsson and Saleh, 2017). No statistically significant differences have been found between Nivolumab and Pembrolizumab, although Nivolumab presents a slight improvement in terms of median overall survival (Moser et al., 2020). Attempts to reduce toxicity through the combination of Nivolumab and Ipilimumab in reduced doses resulted in greater efficacy, but less tolerability than monotherapy (Karlsson and Saleh, 2017; Turajlic et al., 2018; Moser et al., 2020). Nevertheless, most patients still fall in the non-responder-to-ICIs category and many experience immune-related adverse events (irAE), suggesting that despite their potential, resistance and toxicity continue to be their major hurdles.

The lack of ICI response in melanoma patients may be due to different immunological reasons, such as the absence or exclusion of T cells within the tumor microenvironment (TME) or insufficient antigen presentation and priming. Combinations of strategies that turn ICI resistant tumors into responders are being pursued, but, in addition to immunotherapies that directly aim to activate the immune system, all therapeutic strategies would potentially offer some degree of immune activation as well, either by inducing immunogenic cell death of tumor cells and providing antigenic supplies to antigen-presenting cells (APCs), or by triggering inflammatory pathways that will set the tone of the microenvironment towards a possible antitumor response (Chen and Mellman, 2013). Thus, the most obvious way to tackle resistance and perhaps reduce toxicity is through the combination of currently approved therapies.

Recently, studies in melanoma have focused on combining targeted therapies such as BRAF and MEK inhibitors with ICIs, aiming to increase efficacy by tackling resistance; the biology behind this strategy is to promote immune changes within the TME, particularly the release of cancer cell antigens and antigen-presentation by APCs to T cells in a context of checkpoint inhibition, which in turn lead to the activation of effector, tumor-specific T cell clones (Chen and Mellman, 2013). The idea of the strategy is to combine the rapid and deep response of targeted therapy with the durable response of ICIs (Kim et al., 2014; Dummer et al., 2020). *In vivo* melanoma models have shown tumor growth delay, reduced tumor size and prolonged overall survival with the combination of anti-PD-1/anti-PD-L1 and BRAF and MEK inhibitors (Dummer et al., 2020). Currently, there are two studies in clinical phase III, IMspire150 to assess the combination of Atezolimumab (anti-PD-L1) and vemurafenib (BRAF inhibitor), and COMBI-I part 3, assessing the combination of Spartalizaumab (anti-PD-1) plus dabrafenib (BRAF inhibitor) and trametinib (MEK inhibitor); both studies are ongoing. Still, in terms of timing of administration, it is debatable if targeted therapy should be administered before, at the same time or after ICIs; although, the changes induced in the microenvironment by BRAF inhibitors including reducing immunosuppressive cytokines, increasing availability of tumor antigens and infiltration of tumors by immune cells may sensitize tumors to ICIs (Dummer et al., 2020).

Due to the important role that checkpoint molecules play in the homeostasis of the immune system, the intravenous administration and systemic action of ICIs are known to induce undesired irAE. A variety of inflammatory and autoimmune events, ranging from grade 1 to 4 toxicities, have been observed as a result of ICI usage; the most prevalent are the dermatologic toxicities (from grade 1 to 2 rash, pruritus, vitiligo, dermatitis to grade 3 to 4 Stevens-Johnsons syndrome and epidermal necrolysis), present in 50% of melanoma patients treated with anti-CTLA-4 and up to 40% for anti-PD-1 and anti-PD-L1. Other less frequent, but not less relevant, irAEs include gastrointestinal toxicities, from diarrhea to severe colitis, hepatitis with or without elevation of transaminases or bilirubin and fulminant hepatitis; endocrinopathies that include a range of thyroid and pituitary toxicities, adrenal insufficiency and type I diabetes; neurologic toxicities such as autoimmune encephalitis, myasthenia gravis and Guillain-Barré syndrome; renal toxicities include hematuria and acute interstitial nephritis and lupus-like nephritis; ocular toxicity such as uveitis, ulcerative keratitis and retinopathy; cardiac toxicities such as myocarditis, pericarditis, fibrosis, arrhythmias and heart failure and finally, hematological toxicities including hemolytic anemia, thrombocytopenia and eosinophilia (El Osta et al., 2017; Calvo, 2019; Kennedy and Salama, 2019). Despite the increased efficacy, the occurrence of irAEs is indeed more frequent with combinations of different ICIs (Li X. et al., 2017; El Osta et al., 2017; Karlsson and Saleh, 2017; Turajlic et al., 2018). As expected, irAE clinical manifestations are mostly managed with steroids or immunosuppressants, that when used carefully, ameliorate irAE without compromising ICIs efficacy (Karlsson and Saleh, 2017; Calvo, 2019). Even though the possibility of the occurrence of a grade 3 to 4 or chronic irAE is rare, future studies must consider the importance of segregating, through biomarkers, which patients will actually benefit from ICI therapy. Unfortunately, to date, the ideal biomarker for the indication of immunotherapy has not yet been identified (Jessurun et al., 2017). Recent studies in murine melanoma models have suggested a pivotal role of the gut microbiome for ICI efficacy, showing that the presence of some bacterial populations may be associated with increased response to ICIs, while others may be associated with the lack of response (Sivan et al., 2015; Vétizou et al., 2015). Theoretically, through the modulation of microbial populations, non-responders could be turned into responders and perhaps even become less susceptible to ICI toxic effects. In addition, microbial populations may comprise potential biomarkers of ICIs response (Vetizou and Trinchieri, 2018).

Still, there is a clear need for more effective and less toxic strategies. Other checkpoint molecules such as LAG-3, TIM-3, TIGIT, VISTA and B7-H3 and other TME molecules such as IDO have been demonstrated to be promising targets for melanoma and other advanced solid tumors in preclinical studies, granting their passage into clinical trials, although all of them are currently ongoing (Kwiatkowska et al., 2019; Qin et al., 2019). The most studied target, with more than 60 open clinical trials, is the LAG-3 molecule; after binding MHC-II molecules or fibrinogen-like protein 1 (FGL1), LAG-3 restrains the activation, proliferation and cytokine production capacity of Th1 cells while contributing to the suppression activity of Tregs (Qin et al., 2019; Murciano-Goroff and Warner, 2020). In an ongoing phase 1/2 study, anti-LAG-3 antibody (Relatlimab) as monotherapy or in combination with anti-PD-1 is being tested for melanoma patients who were resistant to classical ICIs; early results suggest that the combination is safe and can even increase the antitumor activity of anti-PD-1 alone in ICI-resistant melanoma patients (Kwiatkowska et al., 2019).

Stage IV metastatic melanomas have also been subjected to a personalized strategy developed by Steven Rosenberg at the NIH, resulting in outstanding outcomes and durable complete responses in a few melanoma patients (Prickett et al., 2016). From the lessons learned with IL-2 immunotherapy, Dr. Rosenberg recognized the potential of expanding functional T cells while circumventing the toxicity induced by IL-2 systemic administration, through the *ex vivo* activation with IL-2 of tumor infiltrating lymphocytes which, upon reinfusion, can then target tumor neoantigens (Rosenberg, 2011; Lu et al., 2014; Rosenberg, 2014; Prickett et al., 2016). Despite the great potential of this kind of strategy, known as adoptive cell therapy (ACT), the sophisticated methods required could hamper their inclusion in the clinical routine.

Other strategies that trigger important pathways of the innate immune response have been recognized in preclinical models for their importance in setting the tone of tumors for antitumor responses. Different agonists that trigger innate receptors and sensors such as TLR, STING, RIG-1 and NLR are currently being developed and some, such as STING agonists, have been considered in clinical trials as ICI adjuvants for advanced melanoma and other solid tumors (Hu and Li, 2020); the activation of these receptors is intended to mimic the immune response against viruses, that ultimately trigger cytokines and chemokines that will break the suppressive TME and allow the infiltration of immune cells inside tumors (Clavijo-Salomon et al., 2017). Consistent with the idea of harnessing innate receptor agonists and antiviral responses to fight tumors, oncolytic viruses have demonstrated tremendous potential for the treatment of melanoma, since in addition to awakening antiviral immunity, they can also directly kill tumor cells.

## Using Engineered Viruses for Cancer Immunotherapy

**FIGURE 2:**
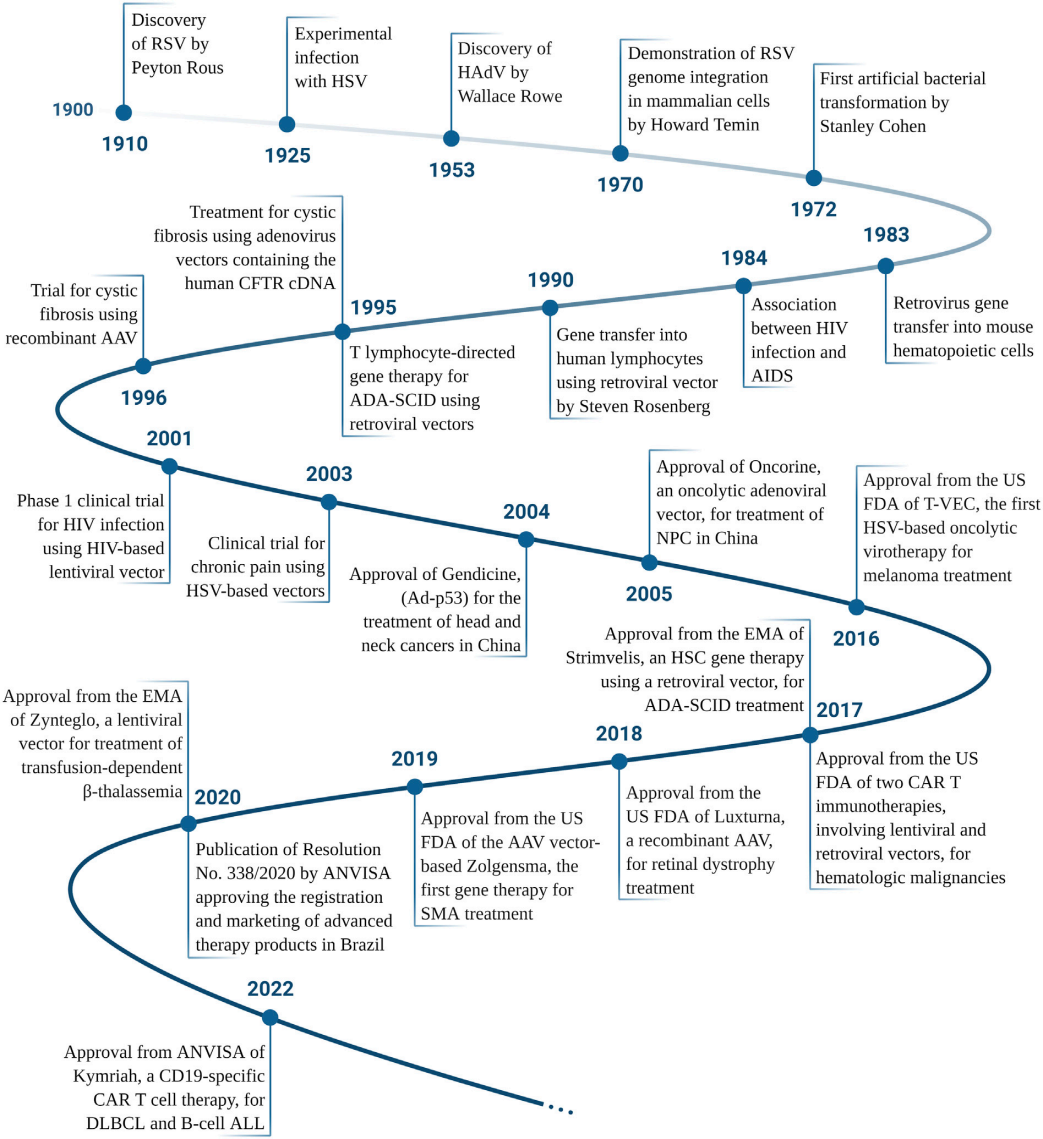
Timeline of the main achievements in the gene therapy and virotherapy fields, according to the date of publication. RSV, Rous Sarcoma Virus; HSV, Herpes Simplex Virus; HAdV, Human adenovirus; HIV, Human Immunodeficiency Virus; AIDS, Acquired Immunodeficiency Syndrome; ADA-SCID, Adenosine Deaminase Severe Combined Immunodeficiency; CFTR, Cystic Fibrosis Transmembrane Conductance Regulator; AAV, Adeno-Associated Virus; NPC, nasopharyngeal carcinoma; US FDA, The United States Food and Drug Administration; EMA, European Medicines Agency; CAR, Chimeric Antigen Receptor; SMA, Spinal Muscular Atrophy; ANVISA, Agência Nacional de Vigilância Sanitária (National Agency for Sanitary Vigilance, the federal body in Brazil that regulates new drugs, among other health related items); DLBCL, Diffuse Large B-Cell Lymphoma; ALL, Acute Lymphoblastic Leukemia. Please see Supplementary Table S1 for references. Created with BioRender.com.

**FIGURE 3:**
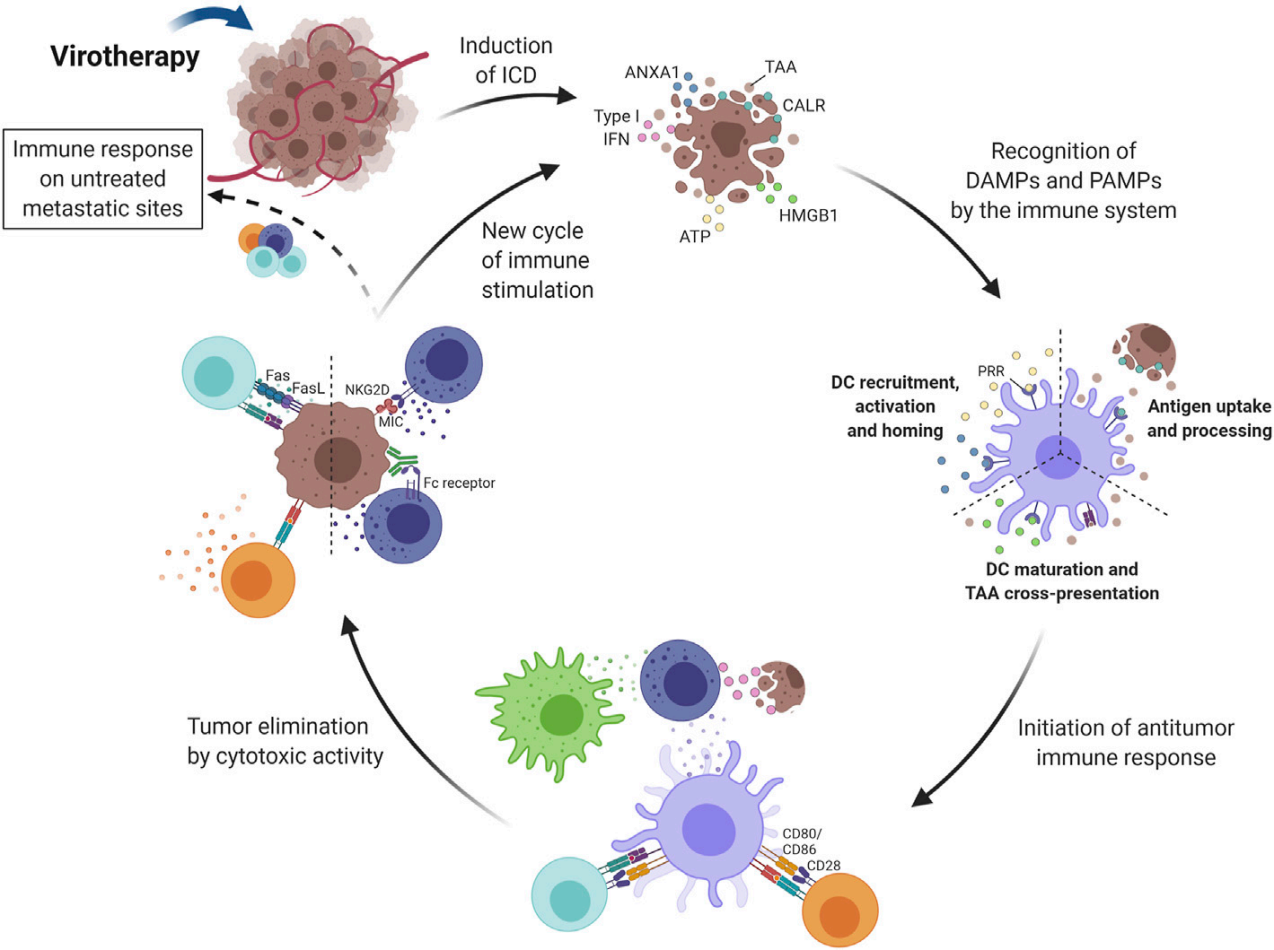
Induction of immune activity in response to virotherapy. Virotherapy can induce ICD, which is characterized by the release of DAMPs (i.e., CALR, HMGB1, ANXA1 and type I IFN) during cell death. These danger signals, along with TAAs and PAMPs also released by OV-treated tumor cells, promote the initiation of an immune response after recognition by antigen-presenting cells (mainly DCs). ATP and ANXA1 are responsible for DC recruitment, activation and homing; CALR increases antigen uptake and processing; and HMGB1 promotes DC maturation and antigen cross-presentation. Next, TAAs are presented to T lymphocytes that can differentiate into both helper and cytotoxic cells since presentation occurs by class I and II MHC proteins. The cytotoxic activity of NK cells is also important for tumor elimination and type I IFNs play an important role in their stimulation together with signals provided by activated DCs and macrophages. Activated effector cells are then capable of recognizing and eliminating tumor cells by different mechanisms, for example, Fas-FasL interaction with CD8^+^ T lymphocytes and MHC recognition by the NKG2D receptor on NK cells. Another key advantage of activating the immune system relies on the possibility of reaching untreated metastatic sites through circulating immune cells. The cycle restarts as dying tumor cells release more antigens and intracellular molecules that keep on activating the immune system. DC, Dendritic cell; NK, Natural killer; T_reg_, Regulatory T cell; ICD, Immunogenic Cell Death; TAA, Tumor-Associated Antigen; CALR, Calreticulin; HMGB1, High Mobility Group Box-1; ATP, Adenosine Triphosphate; IFN, Interferon; ANXA1, Annexin A1; DAMPs, Damage-Associated Molecular Pattern; PAMPs, Pathogen-Associated Molecular Pattern; PRR, Pattern Recognition Receptor; NKG2D, Natural Killer Group 2D; OV, Oncolytic Virus; MHC, Major Histocompatibility Complex. Created with BioRender.com.

The continued study of viruses has brought countless advances not only to the understanding of the molecular basis of diseases, such as cancer itself, but also perspectives for its use as a genetic and therapeutic tool (see Figure 2 and Supplementary Table S1 for key publications in the development of gene therapy), including in Brazil, the first Latin American country with a defined regulatory process for the registration of advanced therapy products. Intriguingly, on one hand viruses that trigger cancer have been discovered (Rous, 1910), yet on the other it has been suggested that some viral infections could improve clinical outcomes for some patients with different types of cancer (Hoster et al., 1949; Newman and Southam, 1954). Though unthinkable today, in 1949 Herman A. Hoster and coworkers used, deliberately, wild type hepatitis B virus in clinical trials of patients with Hodgkin’s disease. In this study, 21 patients were intentionally exposed to the hepatitis virus and 4 of them had improvement in the clinical course, at least with regard to Hodgkin’s. (Hoster et al., 1949). This study was one of the first to intentionally use viral activity to alter the progression of cancer. As presented below, current approaches use a deeper understanding of viral properties, the molecular basis of cancer as well as recombinant DNA techniques in order to use viruses as anti-cancer agents, an approach known as virotherapy or oncolytic viruses (Figure 3). The term “oncolytic viruses” (OV) is typically used to describe genetically modified viruses that selectively infect cancer cells inducing their death, theoretically, without affecting non-malignant tissues (Vähä-Koskela et al., 2007; Russell et al., 2012). Some viruses offer oncolytic properties naturally and do not require modification, for example, Vesicular stomatitis virus, Myxoma, Reovirus, and Newcastle disease virus (Roberts et al., 2006; Kelly and Russell, 2007; Jhawar et al., 2017). Although oncolytic viruses may enter normal cells, progression of the viral life cycle should be inhibited due to molecular components that block viral replication. In tumor cells, many of these mechanisms are dysfunctional or have been suppressed during tumor progression and thus provide a selective advantage for replication and dissemination of viral progeny (Kaufman et al., 2015). For example, resistance to apoptotic cell death, a critical hallmark of cancer, implies that tumor cells are lacking in a fundamental anti-viral defense, a point that may be exploited in order to promote viral replication (Russell et al., 2012). The interferon pathway was originally identified due to its anti-viral properties and, as mentioned above, plays an essential role in inducing innate and adaptive immune responses. While normal cells can defend themselves from viruses using the interferon pathway, tumor cells frequently present deficiencies in interferon response. Thus, this characteristic of tumor cells can be deliberately exploited for the development of OV (Murira and Lamarre, 2016; Gessani and Belardelli, 2021). Tumor cell killing in response to virotherapy occurs due to virus replication and induction of anti-viral responses. As we will detail below, the anti-viral response, which includes activation of innate and adaptive immunity, may be just as important, if not more so, than viral replication.

Our understanding of anti-viral and immunostimulatory properties of both normal cells and neoplasms advanced in the late 1980 s with the discovery of Toll like receptors (TLRs), and later families of Nod (nucleotide-binding and oligomerization domain) -like receptors (NLRs) (Takeda and Akira, 2004; Hansson and Edfeldt, 2005; Inohara et al., 2005). TLRs are present in antigen-presenting cells, such as DCs, macrophages and B cells, as well as T cells, NK cells, and non-immune cells (epithelial and endothelial cells, and fibroblasts), and recognize pathogen-associated molecular patterns (PAMPs), present in both viruses and bacteria, which attract and activate other cells that mediate adaptive immune responses (Fitzgerald and Kagan, 2020). These receptors provided evidence for understanding Coley’s strategy (Coley’s Toxin) (Coley, 1991) and the successful application of BCG in cases of bladder cancer (Lamm et al., 1980), both containing bacterial PAMPs, as well initial works that explored adjuvant effects of viral preparations or natural infections (Dock, 1904; Hoster et al., 1949; Newman and Southam, 1954). In the case of OV, the vector itself provides PAMPs in the form of viral proteins, DNA and RNA that are detected by the cell and initiate the anti-viral cascade through TLRs and NLRs.

In addition, viral infections naturally trigger danger-associated molecular patterns (DAMPs), stress-signaling proteins and inflammatory cytokines (Matzinger, 2002; Tang et al., 2012). As a consequence, strategies employing viruses can induce immunogenic cell death (ICD) of cancer cells, generating chemo-attractants for cells of the immune system (Naik and Russell, 2009). Considering the characteristics of the tumor microenvironment, the use of these oncolytic strategies has the possibility of reversing the immunosuppressive profile and promoting the presentation of the repertoire of tumor antigens in an immunostimulatory context (Bartlett et al., 2013). Besides that, oncolysis triggered by viral particles, replicative or not, has the potential to aggregate immune responses against viral proteins and subvert this for antitumor immunity. When viral systems with replicative capacity are used, tumor selectivity gives these cells new viral epitopes, in addition to the TAAs and/or TSAs. This increases the exposure of these cells to both innate and adaptive immune responses, which may break the vicious cycle of tumor immunoediting. This is the main difference between oncolysis triggered by viral vectors and the approaches outlined above, and for this reason oncolytic virotherapy provides additional advantages over existing therapies that trigger ICD.

As shown in Table 1, several clinical trials have been performed using OV for the treatment of melanoma and novel approaches are being developed. Melanoma was the first neoplasm for which an oncolytic virus therapy was registered by the U.S. Food and Drug Administration (FDA). Approved in 2015, T-VEC, also known as Imlygic (OncoVex, talimogene laherparepvec) is prescribed for patients with advanced melanoma (Stage IIIB, IIIC or IV) that cannot be completely removed with surgery (Fukuhara et al., 2016). The history of this virotherapy exemplifies the path of a new biotechnological tool, from its conception in basic science to clinical trials aimed at proving its safety and efficiency for use in humans. Based on a modified herpes simplex virus (HSV-1), T-VEC was engineered deleting ICP34.5 and ICP47 viral genes. ICP34.5 blocks a cellular stress response to viral infection promoted by IFN-γ and ICP47 impairs the immune system’s CD8 T-cell response against infected cells, thus these viral components render normal cells susceptible to HSV replication. In T-VEC, the deletion of ICP34.5 and ICP47 prevents replication in normal cells, but tumor cells support viral replication due to defects in specific cellular pathways. The deletion of ICP47 also leads to upregulation of the viral protein US11, which further propels virus replication (Goldsmith et al., 1998; Liu et al., 2003). In addition, a constitutive expression cassette was inserted to provide granulocyte-macrophage colony-stimulating factor (GM-CSF) that, in conjunction with other cytokines, contributes to the attraction, differentiation and activation of APCs, such as DCs and macrophages, in the treated areas. Moving forward to phase I clinical trials, T-VEC was well tolerated and caused only mild adverse events such as local erythema and fever (Hu et al., 2006). Next, phase II clinical trials were realized in 50 patients with melanoma, stages III and IV, revealing a 26% response rate, including 8 with complete remission and another 5 with positive partial responses (RECIST) (Senzer et al., 2009). Finally, approval by the FDA and EMA was granted after an open-label phase III study that demonstrated the higher durable response rate (DRR) with a positive impact on overall survival compared to appropriate controls (Andtbacka et al., 2015). Kaufman and collaborators demonstrated that treatment with T-VEC induced a weakening of T cells responsive to MART-1 (melanoma-associated antigen) and, concomitantly, there was a decrease in regulatory T lymphocytes (Kaufman et al., 2010). Intriguingly, T-VEC is administered intratumorally, virus spread is only local, but immune response can mediate tumor regression in non-treated foci. This leads us to question the importance of viral replication itself *vs*. the induction of antitumor immunity for the success of the modality. Another point to be debated is if viral epitopes would indeed be needed for the effectiveness of these immune responses to contain and eliminate the primary tumor, as well metastases.

**TABLE 1:** Clinical trials for treatment of melanoma with oncolytic viruses.

Oncolytic Vector	System	Transgene Load//Vector Main Modifications	Selectivity	Study Phase	Combination	Ref./Number clinical trial
T-VEC (Imlygic, talimogene laherparepvec)	Herpes simplex (HSV-1)	GM-CSF//Deletion: ICP34.5 (blocks PKR-eIF2 pathway) and ICP47 (reduces immune activation) genes	Replication in cells with low protein kinase R (PKR) levels	I, II, III, Approved^a^	—	Conry et al. (2018)
				Ib	Pembrolizumab	Ribas et al. (2017)
				Ib/III	Pembrolizumab	NCT02263508
				Ib/II	Ipilimumab	Chesney et al. (2018)
Pexa-Vec (JX-594, pexastimogene devacirepvec)	Vaccinia	GM-CSF and β-galactosidase//Deletion: thymidine kinase gene (promotes DNA synthesis)	Replication in cells high cellular thymidine kinase activity and active EGFR signaling	Ib/II	Anti-PD-L1 mAb (ZKAB001)	NCT04849260
Telomelysin (OBP-301)	Adenovirus	E1A and E1B regions under control of the human telomerase reverse transcriptase (hTERT) promoter	Replication in cells with telomerase activity	I	—	Nemunaitis et al. (2010)
TILT-123	Adenovirus	IL2 and TNF-α//D24 deletion in the E1A gene (inactivates pRB); E2F promoter	Replication in cells with high expression of E2F and with a dysregulated retinoblastoma pathway	I	TILs	NCT04217473
ICOVIR-5	Adenovirus	D24 deletion in the E1A gene (inactivates pRB); human E2F-1 promoter	Replication in cells with high expression of E2F and with a dysregulated retinoblastoma pathway	I	—	García et al. (2018)
LOAd703 (delolimogene mupadenorepvec)	Adenovirus	4-1BBL and TMZ-CD40L//D24 deletion in the E1A gene (inactivates pRB)	Replication in cells with a dysregulated retinoblastoma pathway	I/II	Atezolizumab	NCT04123470
ONCOS-102 (Ad5/3∆24 GMCSF, CGTG-102)	Adenovirus	GM-CSF//D24 deletion in the E1A gene (inactivates pRB)	Replication in cells with a dysregulated retinoblastoma pathway	I	Pembrolizumab, cyclophosphamide	NCT03003676
GEN0101 (HVJ-E; TSD-0014)^b^	Hemagglutinating virus of Japan (HVJ)	RNA fragmentation by UV irradiation (inactivation); envelope presents fusion activity	Apoptosis and type-1 IFN response mediated by retinoic acid-inducible gene-I (RIG-I) activation in tumor cells upon viral RNA recognition	I/IIa	—	Kiyohara et al. (2020)
				Ib/II	Pembrolizumab	NCT03818893

a by the U.S. FDA, Food and Drug Administration.

b Non-replicating vector.

EGFR, epidermal growth factor receptor; GM-CSF, Granulocyte Macrophage Colony-Stimulating Factor; IFN, interferon; IL2, Interleukin 2; TNF-α, tumor necrosis factor alpha.

Oncolytic viruses have also been developed based on other viral systems. Pexa-Vec (JX-594) is derived from vaccinia virus inactivated by the deletion of the thymidine kinase gene, and modified for the expression of GM-CSF and β-galactosidase transgenes, is in the clinical testing phase for colorectal cancer and hepatocellular carcinoma (Breitbach et al., 2015; Park et al., 2015). A phase I/II clinical trial suggests that intratumoral injection of Pexa-vec is safe and with promising results being effective in treating both injected and distant disease in patients with surgically incurable metastatic melanoma (NCT00429312) (Mastrangelo et al., 1999). Some clinical trials testing Pexa-vec for melanoma are in progress (NCT04849260, NCT02977156). These findings reinforce interest in the use of OV as an immunotherapeutic for melanoma.

Adenovirus is another viral system widely used in immunotherapy for cancer. Oncorine (Onyx-015, H101), for example, is an oncolytic adenovirus-based used for the treatment of head and neck squamous cell carcinoma (Liang, 2018). It was designed to be replicated only in cells that have lost p53 activity, which would cover a large percentage of human neoplasms (Wei et al., 2018). Oncorine was approved in 2005 by State Food and Drug Administration, China (SFDA) (Wei et al., 2018). Although clinical trials of Oncorine for human melanoma have not yet been performed, Hu and colleagues found evidence that the use of ZD55-IL-24 (similar to Oncorine) in an animal model of melanoma prevents tumor growth and induced systemic antitumor immunity (Hu et al., 2020).

Telomelysin (OBP-301) is an oncolytic adenovirus utilizing the human telomerase reverse transcriptase (hTERT) promoter to control the expression of E1A and E1B, key genes that regulate adenoviral replication. In normal cells, the hTERT promoter should not be active, thus the lack of E1A/E1B prevents viral replication. Since hTERT is generally over active in tumor cells, E1A/E1B will be expressed, thus providing selectivity of virus replication (Trager et al., 2020). A phase I clinical trial showed good tolerability, with patients presenting only mild symptoms (grades 1 and 2), such as pain, induration, fever, and chill, and none of them had severe symptoms (grades 3 and 4). Despite having a small cohort, the results were promising, with seven of the twelve patients fulfilling RECIST criteria for stable disease at 56 days after the treatment (Nemunaitis et al., 2010; Trager et al., 2020). In 2016, a phase II clinical trial was initiated testing Telomelysin in patients with unresectable stage III and IV melanoma, though results are not yet available (NCT03190824). In addition, several phase I clinical trials involving replicative adenoviral vectors for different types of cancer have already been carried out, such as TILT-123, ICOVIR-5, LOAd703, ONCOS-102, as shown in Table 1.

As mentioned above, the induction of ICD is essential for the success of oncolytic approaches, bringing into question the importance of virus replication to achieve this goal. Many approaches are being developed for the induction of oncolysis even when the virus, such as adenovirus, does not replicate (Tessarollo et al., 2021). Our research has focused on the use of non-replicating adenoviral vectors for the transfer of genes intended to induce both cell death and immune activation including reversal of the immunosuppressive TME. That is to say, our approach induces oncolysis without the need for a replicating vector. In the first instance, the objective of the gene transfer is to induce immunogenic cell death in cancer cells, and subsequently, a second wave of death due to cytotoxicity mediated by cells of the immune system, mainly T and NK cells. Evidence from our studies indicates that the combined use of interferon-β (IFNβ) and p19Arf (alternate reading frame, p19Arf in mice and p14ARF in humans) induces melanoma cell death by necroptosis and is associated with an anti-viral response and the release of immunogenic factors (such as HMGB1, ATP and calreticulin) (Merkel et al., 2013; Ribeiro et al., 2017; Cerqueira et al., 2020). In our pre-clinical models, this therapy was well tolerated in animals where no side effects, such as liver transaminase induction, were observed and showed promising results in inhibiting tumor growth in s.c. tumors after *in situ* gene therapy, as well as prolonging the survival of treated animals (Cerqueira et al., 2020; David et al., 2020). We have also confirmed the induction of an antitumor immune response in vaccine and immunotherapy settings, with critical involvement of NK cells, CD4^+^ and CD8^+^ T cells, when our vector is used in immunocompetent C57BL/6 mice and B16-F10 mouse melanoma cells (Medrano et al., 2016). When using the human melanoma cell line SK-MEL 147 we demonstrated that transduction with adenoviral vectors encoding p14ARF and IFNβ resulted in activation of monocyte-derived DCs (Cerqueira et al., 2020). In turn, these promoted the activation and priming of T cells, as well as the pro-inflammatory cytokine profile (Cerqueira et al., 2020). Together, these studies show that our gene transfer approach is a promising immunotherapy for melanoma (Hunger et al., 2017; Medrano et al., 2017; Cerqueira et al., 2020). The use of non-replicating vectors may be an advantage for our approach since the delivery of IFNβ can be counterproductive for replicating OVs (Cerqueira et al., 2020; Geoffroy and Bourgeois-Daigneault, 2020; Tessarollo et al., 2021). The results to date are encouraging and research will continue, with critical development using clinically relevant models, such as testing with patient-derived tumor samples, including PDO and immunological *ex vivo* models (Strauss et al., 2018).

## Combining Virotherapy With Different Immunotherapeutic Interventions

**FIGURE 4:**
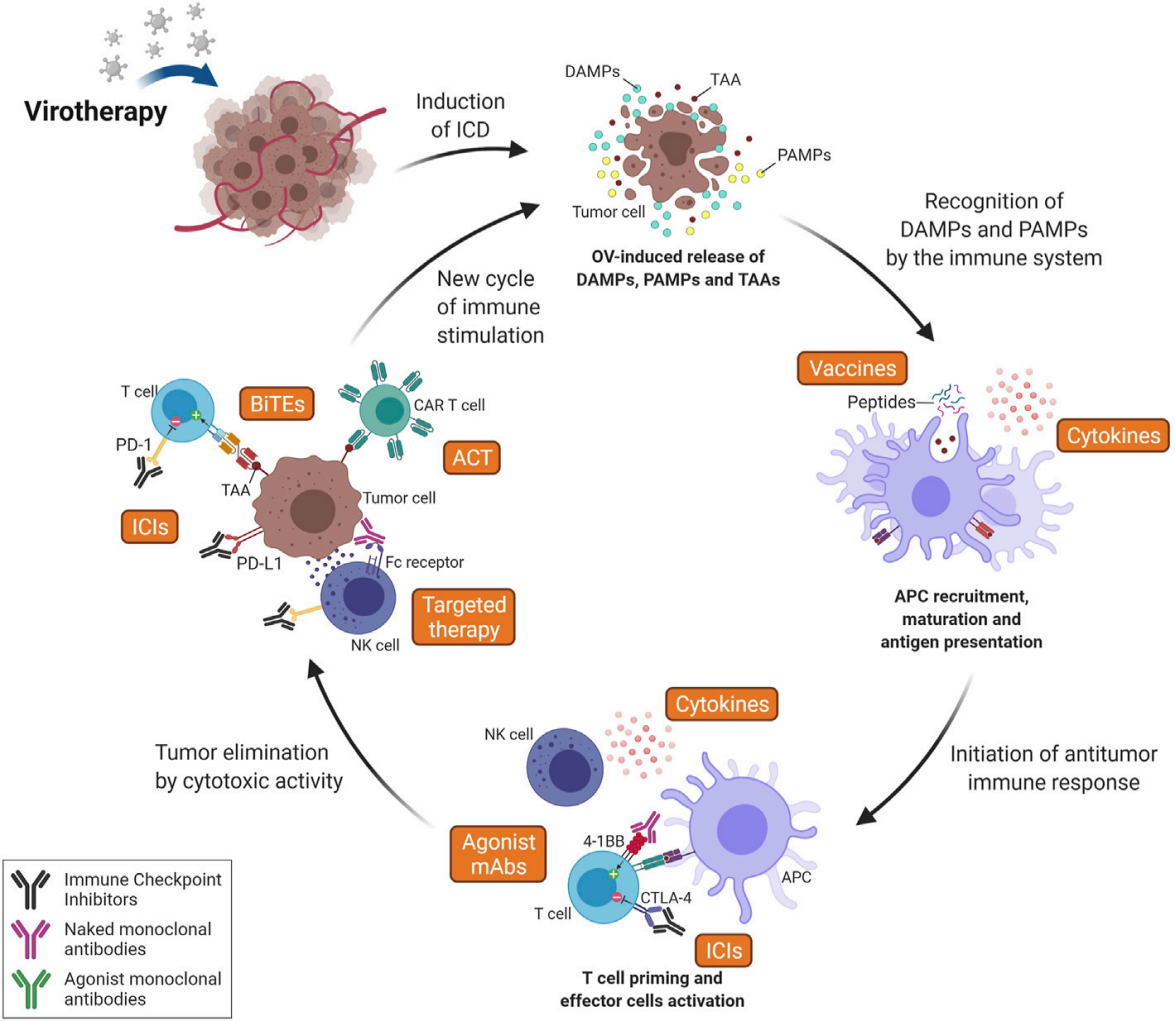
Combining Oncolytic Virus (OV) therapy with other immunotherapy strategies. Since OV acts at the first step of the cancer-immunity cycle, releasing DAMPs, PAMPs and TAAs, its association with additional interventions aiming to establish an antitumor response is favored. The induction of ICD leads to recruitment and activation of antigen-presenting cells (APCs), which can increase the efficiency of vaccine-based approaches (especially peptide vaccines) and generate a stronger T cell response. Immune Checkpoint Inhibitors (ICIs) can increase activation of T cells during priming (here represented by anti-CTLA-4 mAb) and also increase T-cell effector activity in the tumor (i.e., anti-PD-(L)1 mAbs) following OV-induced immune cell infiltration. Agonist monoclonal antibodies (mAbs) are also an interesting intervention to increase T cell activation against TAAs (released after oncolytic therapy) by recognizing and activating costimulatory T cell receptors (i.e., 4-1 BB). Targeted therapy can also potentiate the immune responses to target tumor cells by increasing the effector activity of the innate immune system, including NK cell-mediated ADCC, macrophage-mediated ADCP and complement-mediated CDC. It is important to note that the cytotoxic activity of innate immune cells increases antigen release and, consequently, T cell recruitment. Virotherapy can also increase efficiency of Adoptive Cell Therapy (ACT), here represented by CAR T cells, by promoting a pro-inflammatory microenvironment, enabling T cells enhanced functions and leading to a higher recognition and elimination of tumor cells even in solid tumors. Therapy using bi-specific antibodies, such as BiTEs, can also be enhanced by oncolytic therapy as OVs precondition tumors in terms of T cell recruitment and activation. Finally, cytokines can act at many key steps of the process, such as antigen presentation and T cell priming, activation and recruitment. Furthermore, these factors have an important role at maintaining a favorable microenvironment for the survival of activated immune cells and the sustainment of the immune response. ICD, Immunogenic Cell Death; TAA, Tumor-Associated Antigen; DAMPs, Damage-Associated Molecular Pattern; PAMPs, Pathogen-Associated Molecular Pattern; NK, Natural killer; CTLA-4, Cytotoxic T-Lymphocyte-Associated protein 4; PD-1, Programmed cell Death protein 1; PD-L1, Programmed cell Death Ligand 1; CAR, Chimeric Antigen Receptor; BiTEs, Bi-specific T-cell Engagers; ADCC, Antibody-Dependent Cell-mediated Cytotoxicity; ADCP, Antibody-Dependent Cellular Phagocytosis; CDC, Complement-Dependent Cytotoxicity. Created with BioRender.com.

**FIGURE 5:**
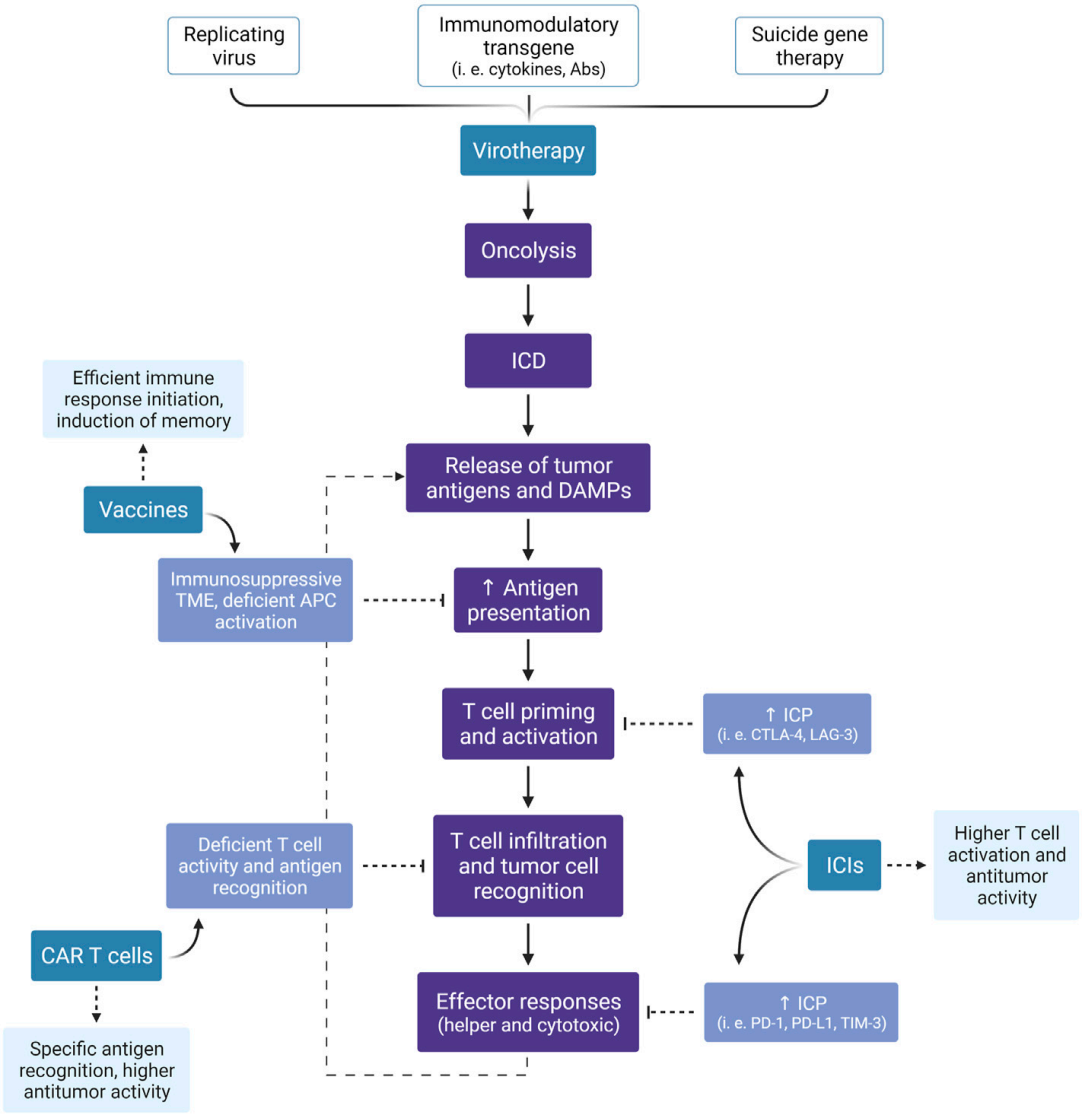
Expected benefits of combined immunotherapies. Besides the direct antitumor effect of virotherapy, especially by the lytic effect of oncolytic viruses, this treatment modality can induce an anti-cancer immune response through the promotion of ICD. Here, we summarize the events illustrated in Figures 3, 4 and highlight the expected advantage when combining virotherapy with other immunotherapy strategies. ICD, Immunogenic Cell Death; DAMPs, Damage-Associated Molecular Patterns; TME, Tumor Microenvironment; APC, Antigen-Presenting Cell; ICP, Immune Checkpoint; ICI, Immune Checkpoint Inhibitor; CTLA-4, Cytotoxic T-Lymphocyte-Associated protein 4; LAG-3, Lymphocyte-Activation Gene 3; PD-1, Programmed cell Death protein 1; PD-L1, Programmed cell Death Ligand 1; TIM-3, T cell Immunoglobulin and Mucin domain-containing protein 3; CAR, Chimeric Antigen Receptor. Created with BioRender.com.

Since the immune system consists of multiple components that act in an ordered and coordinated manner, immunotherapies that target a single step may not promote the entire cascade of events. Instead, combined approaches, especially those that facilitate different steps in the immune response, may provide an improved clinical outcome. As described above, the role of OV is to induce ICD, but this does not guarantee the effectiveness of the steps that follow, including antigen presentation, T cell priming and cytolytic activity. With the success of immunotherapies that target these points, especially ICI, a wide range of combination therapies is possible, as discussed here and summarized in Figures 4, 5.

In the same way that combinations can enhance therapeutic efficacy, they can also be counterproductive or even intensify unwanted side effects. Therefore, clinical trials are essential to provide critical correlative data that can support the combined use of these new therapeutic options. Despite the importance of pre-clinical assays for the initial proof of concept, the animal models used in this stage are limited, since the main potential is precisely the performance of the human immune system (Bareham et al., 2021; Patton et al., 2021). Macedo and collaborators published a review that provides a current overview of virotherapy clinical trials, as well as the resulting combinations (Macedo et al., 2020). According to what they observed, the majority of clinical trials (62.9%) published between 2000 and 2020 investigated only the action of oncolytic viruses used as monotherapy, while 37.1% where OV was administered in combination with at least one other anti-cancer treatment or medication. Of these combinations, the most frequent were cytotoxic chemotherapy agents, chemotherapy prodrugs and radiotherapy. Only a small fraction (5%) of all clinical tests with oncolytic viruses investigated the combination with other immunotherapies such as ICIs or cytokines (Macedo et al., 2020).

### Inhibitors of Immune Checkpoints

As previously mentioned, melanomas have a high mutational load, which contributes to the generation of neoantigens which can be targeted by the patients’ T cells, but their function is often impeded due to upregulation of PD-1. There is also evidence that OV-based therapies increase infiltration of T cells in the tumor (Ribas et al., 2017). Thus, the combination of these immunotherapies is an interesting option (Chiu et al., 2020; Hwang et al., 2020). In randomized, open-label phase I and II studies, T-VEC combined with ipilimumab (anti-CTLA4) showed significantly greater efficacy compared to ipilimumab alone (Puzanov et al., 2016; Chesney et al., 2018; Trager et al., 2020). Likewise, another phase 1b study using T VEC plus pembrolizumab (anti-PD-1) in advanced melanoma showed that this combination was well tolerated, although some patients had mild side effects such as chills, fatigue and pyrexia. Phase III clinical study is already being conducted to expand clinical information regarding efficacy (Long et al., 2016). The patients who responded to the combination therapy showed an increase in the infiltrated CD8^+^ T cells, as well as an increase in the expression of PD-L1 protein, and thus providing mechanistic evidence for the improvement in the effectiveness of pembrolizumab therapy. In addition, greater expression of IFN-γ was detected in the tumor microenvironment in different cell subpopulations, contributing to a less immunosuppressive context (Ribas et al., 2017).

Several approaches using a variety of OVs and ICIs are in pre-clinical and clinical development. For example using a mouse model of metastatic pulmonary melanoma, it was demonstrated that virotherapy using influenza A viruses (IAVs) combined with ICI resulted in a sustained antitumor efficacy caused by the significant increase in the oncolytic effect (Sitnik et al., 2020). In other lines of evidence, Vijayakumar et al., applied Newcastle disease virus (NDV) in combination with radiotherapy plus checkpoint inhibitors (PD1 or CTLA4 targeted mAbs) induces an abscopal effect in immunocompetent B16-F10 murine melanoma model. These authors also show that recombinant NDV a single-chain variable fragment (scFv) anti-CTLA4 plus radiation was as effective as virus, radiation and systemic anti-CTLA4 in terms of survival benefit (Vijayakumar et al., 2019). An important limitation of the use of ICI therapy is that the majority of patients still fail to respond over time (Sharma et al., 2017; Grote et al., 2020). In this context, Liu and collaborators used therapy with oncolytic virus derived from a strain of alphavirus, M1, and were able to demonstrate refractory tumors were sensitized to subsequent checkpoint blockade by boosting T-cell recruitment and upregulating the expression of PD-L1 (Liu et al., 2020). Another important factor to be considered in cancer therapy is whether the effect of local treatment may also include untreated distant metastases. Often referred to as the abscopal effect, this could be an indication that systemic antitumor immune responses are being activated. Evidence in the literature indicates that this effect was observed experimentally in animal models when Kuryk et al. used oncolytic adenoviruses carrying GM-CSF (ONCOS-102) plus ICI therapy (Kuryk et al., 2019). In this scenario of cooperation and synergy, we could also imagine different combinations with other immune players.

### Cytokines

A significant obstacle to successful immunotherapeutic interventions is the modulation of the TME, which, in addition to supporting tumor growth and dissemination, favors the evasion of antitumor immune responses. Dysfunctional interaction of tumor and stromal cellular components leads to a predominantly anti-inflammatory cytokine profile with interleukin-10 (IL-10) (Seo et al., 2001), transforming growth factor (TGF)-beta and other cytokines (Strauss et al., 2007), produced by immunosuppressive cells, for example, regulatory T cells (Tregs) (Knol et al., 2011). The administration of IL-2 was one of the first reproducible effective human cancer immunotherapies against metastatic melanoma (Rosenberg, 2014). Likewise, IFN-α also showed antitumor activity in animal models, in addition to its antiviral activity initially described (Gresser and Bourali, 1970). After clinical trials, both IL-2 and IFN-α demonstrated only mild clinical benefit when used as monotherapy and are approved by the FDA for use in melanoma (Atkins et al., 1999). However, due to the short half-life of most cytokines, high-dose IL-2 and IFN-α administration may be necessary, a situation that increases the incidence of adverse effects, which can make continued treatment unfeasible (Komenaka et al., 2004; Berraondo et al., 2019).

Viruses can be loco-regional adjuvants if applied intratumorally. In addition to their immunostimulatory properties associated with the release of DAMPs and PAMPs, OVs act by inducing acute localized inflammation, they can disturb the tumor niche through the production of inflammatory cytokines in infected/transduced cells. This implies a change in the pattern of cytokines present in the tumor microenvironment in a way that favors the breakdown of immunological tolerance. Few clinical trials have explored the combination of oncologic viruses and cytokine, IL-2 (Voit et al., 2003) and IFN-α (Macedo et al., 2020). However, instead of administering soluble cytokines directly, several approaches function for the design of recombinant virus oncolytic armed with immune modulators, such as cytokines and chemokines. Once the target cell is transduced, it starts to express the carried genes locally, reducing the systemic adverse effects (De Graaf et al., 2018). A classic example is the T-Vec, which locally induces the expression of GM-CSF (Andtbacka et al., 2015). For this purpose, several approaches, with different viral vectors, were used to express cytokines such as IL-2 (Carew et al., 2001; Bai et al., 2014), IL-12 (Varghese et al., 2006), IL-15 (Niu et al., 2015), IFN-γ (Vigil et al., 2007), IFN-β (Durham et al., 2017; Cerqueira et al., 2020; David et al., 2020), reinforces the potential of combining OV and cytokines for immunotherapy for melanomas (De Graaf et al., 2018).

### Oncolytic Vaccines Use TSA/TAA in Combination With OV

Assuming that TSA/TAA are the main targets of the adaptive immune system, some strategies seek to incorporate these tumor antigens in OVs, referred to as “oncolytic vaccines”, designed to potentiate antitumor immune responses, especially cytotoxic T lymphocytes (Elsedawy and Russell, 2013; Holay et al., 2017). Mulryan and coworkers used engineered vaccinia virus expressing TAA 5T4 (an oncofetal antigen), in animal models of melanoma and showed significant melanoma tumor retardation compared with mice vaccinated with respective controls. Although it is a self-antigen, in this work no autoimmune effects inherent to the treatment were detected (Mulryan et al., 2002). In another work, using the B16-ova mosuse melanoma model, Diaz and coworkers verified an increase in the activation of ova-specific T cells after treatment with vesicular stomatitis virus (VSV) delivering ovalbumin (ova) (Diaz et al., 2007). Other studies went further and studied melanoma cDNA library delivery by the oncolytic viral vector VSV. After using these constructs to treat pre-established melanomas in animal models, remission was observed, which is associated with the ability of mouse lymphoid cells to mount a tumor-specific CD4^+^ interleukin (IL)-17 dependent response (Pulido et al., 2012). Collectively, these findings corroborate the principles of personalization of cancer treatment, since the gamut of potential TAA/TSA epitopes will be inherent in the evolutionary history of tumors (Holay et al., 2017). This strategy will be quite valuable in the not-too-distant future, where tumor genome and transcriptome sequencing data will be increasingly available, and thus, likely to be coupled with viral therapies (Finck et al., 2020).

### Perspectives for Combining OV and CAR-T Cell Therapy for Melanoma

Adoptive transfer of chimeric antigen receptor (CAR)-modified T cells has demonstrated remarkable rates of long-lasting complete remission in patients with hematological tumors (Guedan and Alemany, 2018). The approval by the US Food and Drug Administration (FDA) of Kymriah (tisagenlecleucel) for acute lymphoblastic leukemia (ALL) (Maude et al., 2018) and Yescarta (axicabtagene ciloleucel) designed to treat large B-cell lymphoma (Neelapu et al., 2017), opened unprecedented perspectives for cancer treatment. In general, the CAR-T cell approach involves the *ex vivo* modification of the patients’ own T cells using lentiviral and retroviral vectors to deliver the CAR sequence, followed by expansion and reinfusion in the patient (Simon and Uslu, 2018; Chicaybam et al., 2020). Other applications for the use of CAR T have been approved by the FDA as Abecma (idecabtagene vicleucel) for the treatment of multiple myeloma and its use for autoimmune diseases is already being discussed (Hong et al., 2020).

Despite impressive results reported for hematological malignancies, CAR-T cell therapy in solid tumors has failed to meet expectations (Newick et al., 2017; Dana et al., 2021; Marofi et al., 2021). Unlike hematological malignancies, melanomas, like many solid tumors, do not have well-defined targets for the design of a CAR since the available antigens are often expressed in normal cells, thus promoting off-target cell killing. Adding to that difficulty, expression of possible candidate antigens can vary as tumor immunoediting is a continuous process and may give the targeted cells a selective advantage that results in their escape from the CAR-T cells (Dunn et al., 2002; Poggi et al., 2014). Even so, many studies are underway to use the CAR-T cell approach in melanoma (Table 2), a topic that has been reviewed recently (Soltantoyeh et al., 2021; Uslu, 2021). Currently, data from these clinical trials are not available. The immunosuppressive TME is another barrier that must be overcome if CAR-T cell therapies are to be successful. Melanoma, like other solid tumors, is composed of a complex network containing different cell types, such as fibroblasts, endothelial cells, adipocytes and several cells of the immune system immersed in the extracellular matrix (ECM) (Villanueva and Herlyn, 2008; Simiczyjew et al., 2020). Acting together, they comprise an immunosuppressive microenvironment that promotes evasion of antitumor responses, especially of T effector/cytotoxic lymphocytes (Ruiter et al., 2002; Maibach et al., 2020; Simiczyjew et al., 2020). This also directly affects the recruitment and activity of CAR-T cells that may have reached the tumor sites, suggesting that therapy with CAR-T cells alone will not be sufficient to induce complete responses in melanoma. From this perspective, virotherapy, with its ability to revert immunosuppression and promote infiltration of T cells, is expected to fill in some necessary gaps for the effectiveness of CAR-T cell therapy in solid tumors.

**TABLE 2:** Clinical trials using CAR T-cells for the treatment of melanoma and other solid tumors.

Title	Target Antigen	Cancer	Status	ClinicalTrials.gov Identifier
Autologous CAR-T/TCR-T Cell Immunotherapy for Malignancies	CAR-T/TCR-T cells multi-target including CD19, CD22, CD33, BCMA, CD38, NY-ESO-1, DR5, C-met, EGFR V III, Mesothelin	B-cell Acute Lymphoblastic Leukemia, Lymphoma, Myeloid Leukemia, Multiple Myeloma, Hepatoma, Gastric Cancer, Pancreatic Cancer, Mesothelioma, Colorectal Cancer, Esophagus Cancer, Lung Cancer, Glioma, Melanoma, Synovial Sarcoma, Ovarian Cancer, Renal Carcinoma	Recruiting	NCT03638206
B7H3 CAR T Cell Immunotherapy for Recurrent/Refractory Solid Tumors in Children and Young Adults	B7H3	Pediatric Solid Tumor, Germ Cell Tumor, Retinoblastoma, Hepatoblastoma, Wilms Tumor, Rhabdoid Tumor, Osteosarcoma, Ewing Sarcoma, Rhabdomyosarcoma, Synovial Sarcoma, Clear Cell Sarcoma, Malignant Peripheral Nerve Sheath Tumors, Desmoplastic Small Round Cell Tumor, Soft Tissue Sarcoma, Neuroblastoma, Melanoma	Recruiting	NCT04483778
Gene Modified Immune Cells (IL13Ralpha2 CAR T Cells) After Conditioning Regimen for the Treatment of Stage IIIC or IV Melanoma	IL13Ralpha2	Stage IIIC or IV Melanoma	Recruiting	NCT04119024
MB-CART20.1 Melanoma	CD20	Melanoma (Skin)	Unknown	NCT03893019
CAR T Cell Receptor Immunotherapy Targeting VEGFR2 for Patients With Metastatic Cancer	VEGFR2	Metastatic Melanoma	Terminated	NCT01218867
		Renal Cancer		
A Phase I Trial of T Cells Expressing an Anti-GD2 Chimeric Antigen Receptor in Children and Young Adults With GD2+ Solid Tumors	GD2	melanoma, sarcoma, osteosarcoma, neuroblastoma	Completed	NCT02107963
Administering Peripheral Blood Lymphocytes Transduced With a CD70-Binding Chimeric Antigen Receptor to People With CD70 Expressing Cancers	CD70	Melanoma, Pancreatic, Renal, Ovarian and Breast Cancer	Suspended	NCT02830724
B7-H3-Specific Chimeric Antigen Receptor Autologous T-Cell Therapy for Pediatric Patients With Solid Tumors (3 CAR)	B7-H3	Pediatric Solid Tumor, Osteosarcoma, Rhabdomyosarcoma, Neuroblastoma, Ewing Sarcoma, Wilms Tumor, Adrenocortical Cancer, Desmoplastic Small Round Cell Tumor, Germ Cell Cancer, Rhabdoid Tumor, Clear Cell Sarcoma, Hepatoblastoma, Melanoma, Carcinoma, Malignant Peripheral Nerve Sheath Tumors, Soft Tissue Sarcoma	Not yet recruiting	NCT04897321
Treatment of Malignant Melanoma With GPA-TriMAR-T Cell Therapy	GPA-TriMAR	Melanoma	Recruiting	NCT03649529
C7R-GD2.CART Cells for Patients With Relapsed or Refractory Neuroblastoma and Other GD2 Positive Cancers (GAIL-N)	C7R-GD2	Neuroblastoma, Osteosarcoma, Ewing Sarcoma, Rhabdomyosarcoma, Uveal Melanoma, Phyllodes Breast Tumor	Recruiting	NCT03635632
Autologous T Cells Expressing MET scFv CAR (RNA CART-cMET)	MET scFv	Malignant Melanoma, Breast Cancer	Terminated	NCT03060356

In this context, Wing and coworkers used an oncolytic adenovirus to deliver a Bispecific T-cell Engager (BiTE) targeting a second tumor antigen in order to augment tumor cell recognition by CAR-T cells. Surprisingly, this combination was able to activate new populations of antitumor T cells in addition to the CAR-T cells (Wing et al., 2018). Since these assays were conducted in immunocompromised mice (NSG, NOD/SCID/IL2rγ^−/-^), we hypothesize that in immunocompetent individuals, the chance of generating new cytotoxic T cell clones against neoantigens during oncolysis will be increased. Clinical trials will be needed to evaluate this hypothesis, as well as safety. Even though melanoma was not studied, this work opened perspectives for a strategy for other solid tumors. Recently, Jong and collaborators used the same strategy with different targets, sialylated CD43 × CD3 bispecific T cell engager, and demonstrated that it was not only able to bind to cultured patient-derived melanoma samples, but also reduced tumor outgrowth in grafted mice (De Jong et al., 2021). Other studies have pointed out that the use of OV armed with PD-L1 blocking mini-antibody (Tanoue et al., 2017) or IL12p70 and PD-L1 (Rosewell Shaw et al., 2017), combined with CAR-T cell therapy is more effective for tumor control and prolonged survival when compared to each agent as monotherapy. Another interesting evidence in the literature points to the use of oncolytic viral vectors armed with IL-2 and TNF-α to curtail the progression of the primary tumor, but not its metastases. Intriguingly, combining these viral vectors with CAR-T was able to control the primary tumor, as well as its metastases (Guedan and Alemany, 2018; Watanabe et al., 2018).

An important limitation of the use of OV is the incidence of immunity against viral components, including cells that present these viral antigens. Thus, antibodies may neutralize and inactivate viral particles before they reach their target, a limitation of particular concern for repeated administration of the OV. In an innovative approach, VanSeggelen et al. utilized CAR-T cells to protect the oncolytic virus from the immune system, delivering it only to the tumor niche. Thus, viral oncolysis could attract not only more CAR-T cells in a positive feedback loop, but also other immune cells contributing to antitumor responses (Vanseggelen et al., 2015). Collectively these studies point to the potential of these combinations, which need appropriate clinical trials (Guedan and Alemany, 2018).

## Conclusion

Despite numerous advances in therapies for metastatic melanoma, many patients become refractory and succumb to the disease since they are left without treatment options (Keller and Bell, 2016). In this scenario, the search for innovative therapeutic interventions is urgent. Therapies employing oncolytic viruses, replicative or not, are gaining attention. Some successful examples include orphan drug designation for Pexa-Vec (Breitbach et al., 2015; Park et al., 2015) and Telomelysin (Trager et al., 2020) as well as the approval of T-VEC (Fukuhara et al., 2016) and Oncorine (Liang, 2018) by national regulatory agencies. At a time when millions of people are receiving anti-SARS-CoV2 vaccines based on recombinant viral vectors with few/low side effects reported (Yadav et al., 2021), it reinforces safety and reliability with regard to viral vectors as viewed by regulatory agencies around the world.

The use of virotherapy, including gene transfer with non-replicating viral vectors, has been shown to change the profile of TME acting as an adjuvant (Keller and Bell, 2016). As we can see in the diagram in Figure 3, the use of virotherapy induces oncolysis, at this moment by the direct action of the viral particles. In this first round of cell death, due to the release of DAMPs, such as ATP, HMGB1, type I IFNs and exposure of calreticulin, it is characterized as ICD. At the same time, the release of tumor antigens (TAA and TSA) also occurs along with PAMPs associated with the virus. This is a favorable scenario for the recruitment of APCs that capture these tumor antigens and prime T cells, stimulating the formation of adaptive cellular responses against tumor cells. Likewise, the cytokine profile in the tumor microenvironment tends to change from an immunosuppressive to an inflammatory profile, favoring the recruitment of effector T cells. Thereafter, a second wave of oncolysis begins, but this time due to cytotoxicity of T cell antitumor clones, which can even act in distant metastatic sites with the application of viral therapy.

As novel therapies emerge, rational combinations will need to be overcome tumor resistance and adaptations of tumor cells and their cellular partners in the tumor microenvironment (Raja et al., 2018). Taking advantage of the fact that virotherapy can attract T cells to the tumor niche, ICIs can have their effectiveness enhanced if used together (Figures 4, 5). Clinical trial has already been carried out to envision this combination (Long et al., 2016; Puzanov et al., 2016; Chesney et al., 2018; Trager et al., 2020). By the same reasoning, the use of CAR T cells against solid tumors such as melanoma is expected to be more effective when combined with virotherapy. However, as there is still no registered CAR-T cell therapy for melanoma, clinical trials will be necessary to verify this hypothesis.

Since the tumor microenvironment is abundant in anti-inflammatory cytokines such as IL-10 and TGF-β, the use of armed viral vectors can reverse this profile and restrict the expression of appropriately chosen cytokines, to favor a less immunosuppressive context. Unlike the systemic application of cytokines, which brings together a series of collateral effects, intratumoral expression by viral vectors tends to increase their availability in this microenvironment with a reduction in side effects. In summary, improvements in the design, delivery and targeting of oncolytic viral vectors will provide increasing potential as immunotherapies against melanoma. Allied to this, is the fact that combinations with different immunotherapy modalities can cooperate to increase therapeutic efficacy.
