# Supplementary material for: Perspectives for Combining Viral Oncolysis With Additional Immunotherapies for the Treatment of Melanoma
Source: Front Mol Biosci. 2022 Apr 14;9:777775. doi: 10.3389/fmolb.2022.777775 (PMC9048901; doi:10.3389/fmolb.2022.777775)
Supplement: Supplementary file 1 [file Table1.pdf]

Table S1. References for timeline.

Year	Event	Citation
1910	Discovery of RSV by Peyton Rous	(Rous, 1910)
1925	Experimental infection with HSV	(Goodpasture, 1925)
1953	Discovery of HAdV by Wallace Rowe	(Rowe et al., 1953)
1970	Demonstration of RSV genome integration in mammalian cells by Howard Temin	(Temin and Mizutani, 1970)
1972	First artificial bacterial transformation by Stanley Cohen	(Cohen et al., 1972)
1983	Retrovirus gene transfer into mouse hematopoietic cells	(Joyner et al., 1983)
1984	Association between HIV infection and AIDS	(Broder and Gallo, 1984)[a]
1990	Gene transfer into human lymphocytes using retroviral vector by Steven Rosenberg	(Rosenberg et al., 1990)
1995	Treatment for cystic fibrosis using adenovirus vectors containing the human CFTR cDNA	(Korst et al., 1995)
1995	T lymphocyte-directed gene therapy for ADA-SCID using retroviral vectors	(Blaese et al., 1995)

1996	Trial for cystic fibrosis using recombinant AAV.	(Flotte et al., 1996)
2001	Phase 1 clinical trial for HIV infection using HIV-based lentiviral vector.	(MacGregor, 2001)
2003	Clinical trial for chronic pain using HSV-based vectors	(Glorioso et al., 2003)
2004	Approval of Gendicine (Ad-p53) for the treatment of head and neck cancers in China	(Pearson et al., 2004)
2005	Approval of Oncorine, an oncolytic adenoviral vector, for treatment of NPC in China	(Liang, 2018)
2016	Approval from the US FDA of T-VEC, the first HSV-based oncolytic virotherapy for melanoma treatment	(Greig, 2016)
2017	Approval from the FDA of two CAR T immunotherapies, involving lentiviral and retroviral vectors, for hematologic malignancies	(Ledford, 2017)
2017	Approval from the EMA of Strimvelis, an HSC gene therapy using a retroviral vector, for ADA-SCID treatment	(Aiuti et al., 2017)
2018	Approval from the US FDA of Luxturna, a recombinant AAV, for retinal dystrophy treatment	(Ameri, 2018)
2019	Approval from the FDA of the AAV vector-based Zolgensma, the first gene therapy for SMA treatment	(Hoy, 2019)
2020	Approval from the EMA of Zynteglo, a lentiviral vector for treatment of transfusion-dependent β-thalassemia	(Schuessler-Lenz et al., 2020)
2020	Publication of Resolution No. 338/2020 by ANVISA approving the registration and marketing of advanced therapy products in Brazil (https://www.in.gov.br/en/web/dou/-/resolucao-da-diretoria-colegiada-rdc-n-338-de-20-de-fevereiro-de-2020-244803291)	
2022	Approval from ANVISA of Kymriah, a CD19-specific CAR T cell therapy for the treatment of DLBCL and B-cell ALL (https://www.gov.br/anvisa/pt-br/assuntos/noticias-anvisa/2022/anvisa-aprova-produto-de-terapia-avancada-para-tratamento-de-cancer)	

a, At the time, the HIV virus was known as HTLV-III.

RSV, Rous Sarcoma Virus; HSV, Herpes Simplex Virus; HAdV, Human adenovirus; HIV, Human Immunodeficiency Virus; AIDS, Acquired Immunodeficiency Syndrome; ADA-SCID, Adenosine Deaminase Severe Combined Immunodeficiency; CFTR, Cystic Fibrosis Transmembrane Conductance Regulator; AAV, Adeno-Associated Virus; NPC, nasopharyngeal carcinoma; US FDA, The United States Food and Drug Administration; EMA, European Medicines Agency; CAR, Chimeric Antigen Receptor; SMA, Spinal Muscular Atrophy; ANVISA, Agência Nacional de Vigilância Sanitária (National Agency for Sanitary Vigilance, the federal body in Brazil that regulates new drugs, among other health related items); DLBCL, Diffuse Large B-Cell Lymphoma; ALL, Acute Lymphoblastic Leukemia.

References

Aiuti, A., Roncarolo, M.G., and Naldini, L. (2017). Gene therapy for ADA-SCID, the first marketing approval of an ex vivo gene therapy in Europe: paving the road for the next generation of advanced therapy medicinal products. *EMBO molecular medicine* 9**,** 737-740.

Ameri, H. (2018). Prospect of retinal gene therapy following commercialization of voretigene neparvovec-rzyl for retinal dystrophy mediated by RPE65 mutation. *Journal of current ophthalmology* 30**,** 1-2.

Blaese, R.M., Culver, K.W., Miller, A.D., Carter, C.S., Fleisher, T., Clerici, M., Shearer, G., Chang, L., Chiang, Y., Tolstoshev, P., Greenblatt, J.J., Rosenberg, S.A., Klein, H., Berger, M., Mullen, C.A., Ramsey, W.J., Muul, L., Morgan, R.A., and Anderson, W.F. (1995). T lymphocyte-directed gene therapy for ADA- SCID: initial trial results after 4 years. *Science* 270**,** 475-480.

Broder, S., and Gallo, R.C. (1984). A pathogenic retrovirus (HTLV-III) linked to AIDS. *The New England journal of medicine* 311**,** 1292-1297.

Cohen, S.N., Chang, A.C., and Hsu, L. (1972). Nonchromosomal antibiotic resistance in bacteria: genetic transformation of Escherichia coli by R-factor DNA. *Proc Natl Acad Sci U S A* 69**,** 2110-2114.

Flotte, T., Carter, B., Conrad, C., Guggino, W., Reynolds, T., Rosenstein, B., Taylor, G., Walden, S., and Wetzel, R. (1996). A phase I study of an adeno-associated virus-CFTR gene vector in adult CF patients with mild lung disease. *Hum Gene Ther* 7**,** 1145-1159.

Glorioso, J.C., Mata, M., and Fink, D.J. (2003). Gene therapy for chronic pain. *Curr Opin Mol Ther* 5**,** 483-488.

Goodpasture, E.W. (1925). The Axis-Cylinders of Peripheral Nerves as Portals of Entry to the Central Nervous System for the Virus of Herpes Simplex in Experimentally Infected Rabbits. *Am J Pathol* 1**,** 11-28.15.

Greig, S.L. (2016). Talimogene Laherparepvec: First Global Approval. *Drugs* 76**,** 147-154.

Hoy, S.M. (2019). Onasemnogene Abeparvovec: First Global Approval. *Drugs* 79**,** 1255-1262.

Joyner, A., Keller, G., Phillips, R.A., and Bernstein, A. (1983). Retrovirus transfer of a bacterial gene into mouse haematopoietic progenitor cells. *Nature* 305**,** 556-558.

Korst, R.J., Mcelvaney, N.G., Chu, C.-S., Rosenfeld, M.A., Mastrangeli, A., Hay, J., Brody, S.L., Eissa, N.T., Danel, C., Jaffe, H.A., and Crystal, R.G. (1995). Gene Therapy for the Respiratory

Manifestations of Cystic Fibrosis. *American Journal of Respiratory and Critical Care Medicine* 151**,** S75-S87.

Ledford, H. (2017). Engineered cell therapy for cancer gets thumbs up from FDA advisers. *Nature News* 547**,** 270.

Liang, M. (2018). Oncorine, the World First Oncolytic Virus Medicine and its Update in China. *Curr Cancer Drug Targets* 18**,** 171-176.

Macgregor, R.R. (2001). Clinical protocol. A phase 1 open-label clinical trial of the safety and tolerability of single escalating doses of autologous CD4 T cells transduced with VRX496 in HIV-positive subjects. *Hum Gene Ther* 12**,** 2028-2029.

Pearson, S., Jia, H., and Kandachi, K. (2004). China approves first gene therapy. *Nature Biotechnology* 22**,** 3-4.

Rosenberg, S.A., Aebersold, P., Cornetta, K., Kasid, A., Morgan, R.A., Moen, R., Karson, E.M., Lotze, M.T., Yang, J.C., Topalian, S.L., and Et Al. (1990). Gene transfer into humans--immunotherapy of patients with advanced melanoma, using tumor-infiltrating lymphocytes modified by retroviral gene transduction. *N Engl J Med* 323**,** 570-578.

Rous, P. (1910). A TRANSMISSIBLE AVIAN NEOPLASM. (SARCOMA OF THE COMMON FOWL.). *J Exp Med* 12**,** 696-705.

Rowe, W.P., Huebner, R.J., Gilmore, L.K., Parrott, R.H., and Ward, T.G. (1953). Isolation of a cytopathogenic agent from human adenoids undergoing spontaneous degeneration in tissue culture. *Proc Soc Exp Biol Med* 84**,** 570-573.

Schuessler-Lenz, M., Enzmann, H., and Vamvakas, S. (2020). Regulators' Advice Can Make a Difference: European Medicines Agency Approval of Zynteglo for Beta Thalassemia. *Clinical pharmacology and therapeutics* 107**,** 492-494.

Temin, H.M., and Mizutani, S. (1970). RNA-dependent DNA polymerase in virions of Rous sarcoma virus. *Nature* 226**,** 1211-1213.
